# Risk stratification of papillary thyroid cancers using multidimensional machine learning

**DOI:** 10.1097/JS9.0000000000000814

**Published:** 2023-11-01

**Authors:** Yuanhui Li, Fan Wu, Weigang Ge, Yu Zhang, Yifan Hu, Lingqian Zhao, Wanglong Gou, Jingjing Shi, Yeqin Ni, Lu Li, Wenxin Fu, Xiangfeng Lin, Yunxian Yu, Zhijiang Han, Chuanghua Chen, Rujun Xu, Shirong Zhang, Li Zhou, Gang Pan, You Peng, Linlin Mao, Tianhan Zhou, Jusheng Zheng, Haitao Zheng, Yaoting Sun, Tiannan Guo, Dingcun Luo

**Affiliations:** a Department of Oncological Surgery; j Department of Radiology; k Department of Ultrasonography; l Department of Pathology; m Centre of Translational Medicine, Hangzhou First People’s Hospital; f Key Laboratory of Structural Biology of Zhejiang Province, School of Life Sciences, Westlake University; g Research Centre for Industries of the Future, Westlake University; e Westlake Center for Intelligent Proteomics, Westlake Laboratory of Life Sciences and Biomedicine, Hangzhou, Zhejiang Province; i Department of Epidemiology and Health Statistics, School of Public Health, Zhejiang University; b bWestlake Omics (Hangzhou) Biotechnology Co., Ltd; c The Fourth Clinical Medical College, Zhejiang Chinese Medical University; d Key Laboratory of Growth Regulation and Translational Research of Zhejiang Province, School of Life Sciences, Westlake University, Hangzhou, Zhejiang; h Department of Thyroid Surgery, The Affiliated Yantai Yuhuangding Hospital of Qingdao University, Shandong Province, People’s Republic of China

**Keywords:** machine learning, papillary thyroid cancer, proteomics, risk stratification

## Abstract

**Background:**

Papillary thyroid cancer (PTC) is one of the most common endocrine malignancies with different risk levels. However, preoperative risk assessment of PTC is still a challenge in the worldwide. Here, the authors first report a Preoperative Risk Assessment Classifier for PTC (PRAC-PTC) by multidimensional features including clinical indicators, immune indices, genetic feature, and proteomics.

**Materials and Methods:**

The 558 patients collected from June 2013 to November 2020 were allocated to three groups: the discovery set [274 patients, 274 formalin-fixed paraffin-embedded (FFPE)], the retrospective test set (166 patients, 166 FFPE), and the prospective test set (118 patients, 118 fine-needle aspiration). Proteomic profiling was conducted by FFPE and fine-needle aspiration tissues from the patients. Preoperative clinical information and blood immunological indices were collected. The *BRAF*^V600E^ mutation were detected by the amplification refractory mutation system.

**Results:**

The authors developed a machine learning model of 17 variables based on the multidimensional features of 274 PTC patients from a retrospective cohort. The PRAC-PTC achieved areas under the curve (AUC) of 0.925 in the discovery set and was validated externally by blinded analyses in a retrospective cohort of 166 PTC patients (0.787 AUC) and a prospective cohort of 118 PTC patients (0.799 AUC) from two independent clinical centres. Meanwhile, the preoperative predictive risk effectiveness of clinicians was improved with the assistance of PRAC-PTC, and the accuracies reached at 84.4% (95% CI: 82.9–84.4) and 83.5% (95% CI: 82.2–84.2) in the retrospective and prospective test sets, respectively.

**Conclusion:**

This study demonstrated that the PRAC-PTC that integrating clinical data, gene mutation information, immune indices, high-throughput proteomics and machine learning technology in multicentre retrospective and prospective clinical cohorts can effectively stratify the preoperative risk of PTC and may decrease unnecessary surgery or overtreatment.

## Introduction

Highlights

- The preoperative risk assessment classifier for papillary thyroid cancer (PRAC-PTC) was constructed by multidimensional features to stratify the preoperative risk of PTC.
- Six proteins (DPP7, PDLIM3, Col12A1, CTSL, TUBB2A, and ITGB5) were identified as the best discriminable proteins between low-risk and intermediate-risk /high-risk PTCs.
- The PRAC-PTC improved the preoperative predictive risk effectiveness of clinicians with different seniority levels.

Papillary thyroid cancer (PTC) is one of the most common endocrine malignancies^1,2^. Although the prognosis of PTC is favourable, it tends to have a high rate of recurrence (10–35%), which may lead to treatment failure and death^3^. PTCs can be divided into different risk levels. In recent years, it has been believed that some relatively inert PTCs can be managed with active surveillance without immediate surgery. However, 15–30% of invasive PTCs have a poor prognosis and require active surgical treatment^1,4^. At present, achieving an accurate preoperative risk assessment of PTC to avoid excessive or insufficient treatment remains challenging.

The modified 2015 American Thyroid Association (ATA) management guidelines proposed a three-tiered clinicopathologic risk stratification system that classified PTC patients as either low, intermediate, or high-risk based on structural recurrence^5^. Low-risk PTC patients show no evidence of extrathyroidal extension or small-volume metastases. Intermediate-risk PTC patients have microscopic extrathyroidal extension, clinical N1 disease or cervical lymph node metastases (>5 metastatic lymph nodes or maximum diameter of metastatic focus ≥0.2 cm and <3 cm). High-risk PTC patients are characterised by gross extrathyroidal extension, metastatic lymph nodes greater than or equal to 3 cm, incomplete tumour resection, and distant metastases^5^. PTC patients with different risk levels require distinct treatment strategies. Active surveillance can be implemented for low-risk PTC patients due to the slow progression in active detection^6,7^. Surgery and additional radioiodine therapy are usually required for intermediate-risk and high-risk patients because these PTC tumours progress rapidly and are prone to developing lymph node metastasis or distant metastasis. Therefore, when PTC patients are first diagnosed, clinicians need to evaluate the risk degree objectively. Currently, PTC patients are preoperatively assessed using physical examinations, imaging, genetic testing, and so on. However, these methods fail to achieve accurate stratification based on risk level^8^. Although previous studies have reported the risk stratification and predictors of PTC, including clinicopathological and ultrasound features^9^, lncRNAs^10^, and immune indices^11^, these single-aspect predictors showed low predictive efficiency and were not validated in retrospective and prospective analyses, which decreased the credibility of the findings. Thus, an effective preoperative risk stratification method is needed for treating PTC.

Proteins are the executors of life activities^12–14^, and they also play important roles in the diagnosis and treatment of diseases. In this new era of clinical proteomics, extensive progress has been made in the last few years; some examples include pressure cycling technology (PCT)-based sample preparation with a small amount of sample and formalin-fixed paraffin-embedded (FFPE) sample preparation for proteomics^15^ as well as data-independent acquisition by mass spectrometry (DIA-MS) for large numbers of samples^16^. Artificial intelligence (AI)-empowered big data proteomics has come to facilitate precision medicine. In recent years, an increasing number of studies have employed AI in multiple roles in the field of tumour research, such as in predicting cancer immunotherapy efficacy^12–14^, discovering and developing drugs^17,18^, and exploring molecular mechanisms^19,20^. Molecular data and imaging have leveraged AI to correlate these sources with tumour detection, monitoring of tumour progression, and identification of optimised therapeutic treatment^21^. Our previous study reported an AI-defined protein-based biomarker panel for the diagnostic classification of thyroid nodules that can distinguish between benign and malignant thyroid nodules with an accuracy of 91%, emphasising the superior performance of proteomics in clinical diagnosis^16^. At present, we focus on the biological behaviour of malignant tumours and intend to distinguish low-risk and intermediate-/high-risk PTC before surgery to provide guidance for precision treatment.

In this study, we collected multidimensional features, including clinical, immunological, genetic, and proteomic information, from 558 PTC patients with a machine learning model to differentiate low-risk PTC from intermediate-/high-risk PTC for constructing a Preoperative Risk Assessment Classifier for PTC (PRAC-PTC). Referencing the modified 2015 ATA management guidelines, we classified the risk degree of all enroled patients according to the initial postoperative pathology, and we predicted the possible postoperative pathology and risk of each patient with PRAC-PTC. The predictive efficacy of PRAC-PTC was then validated in retrospective and prospective cohorts. We further explored whether clinicians could improve their ability to preoperatively predict risk effectiveness with the assistance of PRAC-PTC.

## Materials and methods

### Participants

**Figure 1:**
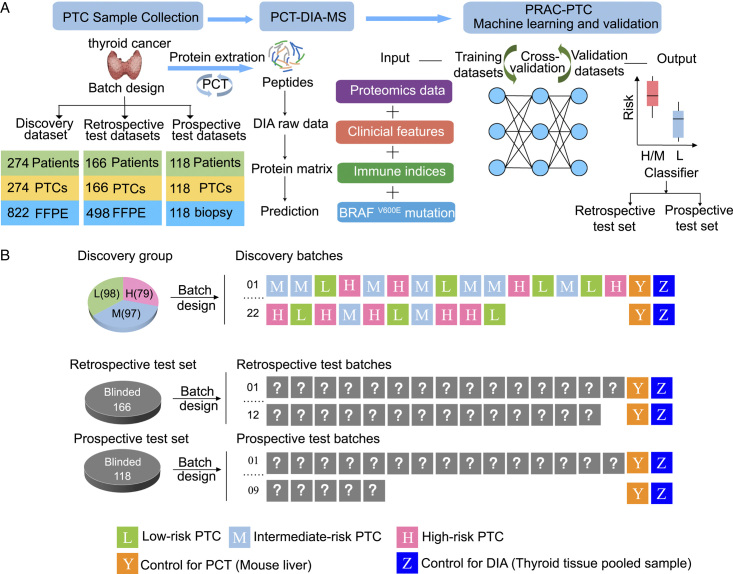
Study workflow. (A) Project design and workflow for the FFPE and FNA PCT-DIA pipeline. (B) Batch design of the discovery group, the retrospective test group, and the prospective test group. The discovery group consisted of 274 PTC samples collected from 274 patients, including 79 high-risk, 97 intermediate-risk, and 98 low-risk PTC samples. A total of 274 PTC and 27 technical replicates were then randomly allocated to 22 batches to minimise the batch effect. The independent test group included the retrospective and prospective test groups. The retrospective test group included 166 PTCs from 166 patients (blinded arrangements) divided into 12 batches for the analysis. Technical duplicates were generated from each retrospective test group sample, but no biological replicates were performed. The prospective test group included 118 FNA biopsies of thyroid nodules from 118 patients divided into nine batches. Each batch consisted of 15 thyroid samples, one mouse liver sample, and one pooled thyroid sample.

A total of 558 patients were enroled from two hospitals in China: Hangzhou First People’s Hospital and Yuhuangding Hospital. The 558 patients were allocated to three groups: the discovery set (274 patients, 274 samples), retrospective test set (166 patients, 166 samples), and the prospective test set (118 patients, 118 samples) (Fig. 1A). For patient recruitment, the inclusion criteria were as follows: (1) primary surgery and lymph node dissection; (2) no previous history of chemotherapy and radiotherapy; (3) a postoperative diagnosis of classic PTC (C-PTC) made by two pathologists; and (4) a postoperative pathological diagnosis containing complete information about the risk stratification. The exclusion criteria were as follows: (1) a history of neck trauma; (2) other concurrent or previous cancers; (3) a postoperative pathological diagnosis of another subtype of PTC or other pathological type; and (4) lack of complete and available postoperative pathology.

The study methodologies complied with the Declaration of Helsinki and were approved by the Research Ethics Committee of each hospital. The research was performed after informed consent was obtained from the patients. This work has been reported in line with the REMARK criteria^22^.

### Clinical data collection

All PTC cases were confirmed by postoperative pathology, and the structural recurrence risk was assessed according to the modified 2015 ATA management guidelines^5^. For machine learning model construction and verification, we collected seven preoperative clinical indicators: sex, age, tumour diameter (based on ultrasonography), Hashimoto’s thyroiditis (based on chemiluminescence detection and ultrasonography), multifocality (based on ultrasonography), capsular invasion (based on ultrasonography), and extrathyroidal extension (based on ultrasonography) (Supplementary Table S1, Supplemental Digital Content 1, http://links.lww.com/JS9/B246). Systemic inflammatory responses are predictive biomarkers and potential treatment strategies in various diseases, including PTC^23–25^. Here, we collected eight blood immunological indices before surgery: platelet counts, neutrophil counts, lymphocyte counts, monocyte counts, platelet-to-lymphocyte ratio, neutrophil-to-lymphocyte ratio, lymphocyte-to-monocyte ratios (LMR), and systemic immune-inflammation index (SII). As the *BRAF*^V600E^ mutation is an independent predictor of the risk of recurrence and is closely linked with a poorer clinical prognosis^26–28^, we also detected the *BRAF*^V600E^ mutation using the amplification refractory mutation system.

### Sample collection and proteomic analysis

The sample collection details are provided in the Supplementary Materials (Supplemental Digital Content 5, http://links.lww.com/JS9/B251). Proteomic sample preparation was performed according to our previously published methods^15,16,29^. Each FFPE tissue block and fine-needle aspiration (FNA) biopsy samples were processed by PCT, and data were acquired by DIA-MS using the settings described in our previous publication. The raw proteomic data were analysed using DIA-NN (v1.7.15) against a thyroid-specific spectral library^30^.

### Exploration of the PTC risk assessment strategy

We explored the PTC risk assessment strategy from three aspects. First, we constructed prediction models with the XGBoost algorithm for each of the increasing groups of feature dimensions and compared their effectiveness. Model A was based on clinical indicators, model B was based on clinical and immunological indices, and model C was based on clinical, immunological, and genetic indices. The model based on four dimensions (including clinical, immunological, genetic, and proteomic indices), namely, the Preoperative Risk Assessment Classifier for PTC (PRAC-PTC), had the best predictive efficacy. The modelling steps are detailed in the Supplementary Materials (Supplemental Digital Content 5, http://links.lww.com/JS9/B251).

Second, we determined the accuracy of nine clinicians with different seniority levels, including three junior clinicians (1–5 years of experience), three medium-level clinicians (5–10 years of experience), and three senior clinicians (>10 years of experience), in preoperatively predicting PTC risk based on ultrasonography and computed tomography (CT). Detailed information on the clinicians’ preoperative risk stratification is shown in the Supplementary Materials (Supplemental Digital Content 5, http://links.lww.com/JS9/B251).

Third, initial independent assessments were made by the nine clinicians alone and then with reference to the PRAC-PTC for the final assessments. The PRAC-PTC outputs a score for each patient; if the score is less than 0.6645 (the ratio of the number of high-/intermediate-risk samples to the number of low-risk samples in the discovery test), the PRAC-PTC considers the case to be low-risk PTC; if the score is more than 0.6645, the PRAC-PTC considers the case to be intermediate-/high-risk PTC. If the two results did not match, the clinicians could then choose either their result or that of PRAC-PTC as the final result. We compared the assessment effectiveness of PRAC-PTC with that of the clinicians and the benefits to the clinicians of PRAC-PTC assistance.

### Statistical analysis

The statistical analyses were performed using R (version 3.5.1). Specifically, we used heatmaps, uniform manifold approximation and projection (UMAP), and R packages for plotting functions. The coefficient of variation (CV) was calculated as the ratio of the SD to the mean. Differentially expressed proteins (DEPs) between each paired groups were analysed using the threshold of *P*<0.05 and fold change >2. *P* values calculated by unpaired two-sided Welch’s t test. Sensitivity, specificity, positive predictive value (PPV), and negative predictive value (NPV) were calculated following the established methodology^31^; each value was calculated with 95% Wilson CIs. Biological insights were analysed by the Metascape web-based platform^32^.

## Results

### Study design and multidimensional features collection

**Figure 2:**
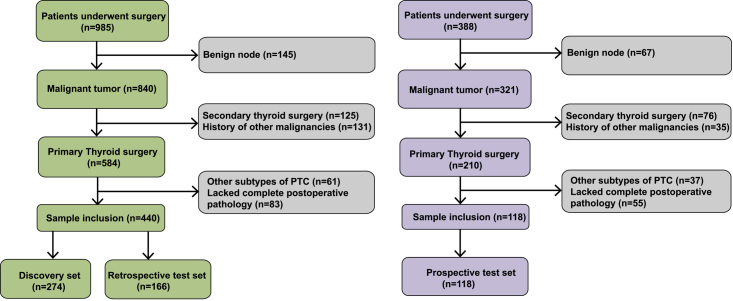
Process of the patient enrolment for discovery set and two independent test sets.

The patient samples from Hangzhou First People’s Hospital and Yuhuangding Hospital were divided into three cohorts: (i) a discovery set of FFPE samples (*n*=274 PTCs), (ii) a retrospective test set of FFPE samples (*n*=166 PTCs), and (iii) a prospective test set of FNA biopsy samples (*n*=118 PTCs) (Fig. 1A). The inclusion and exclusion criteria are described in the Methods section and Figure 2. The clinical risk stratification of the PTC patients was evaluated by clinicians from Hangzhou First People’s Hospital and Yuhuangding Hospital following the American Thyroid Association (ATA) management guidelines^5^. Specifically, in the discovery set, 98 patients were classified as low-risk, 97 as intermediate-risk, and 79 as high-risk (Table 1).

**Table 1:** Baseline characteristics.

Characteristics	Discovery sets (*n*=274)	Retrospective test sets (*n*=166)	Prospective test set (*n*=118)
Total no			
Clinical centres	2	2	2
Patients	274	166	118
PTCs	274	166	118
FFPE cores	819	498	0
Fine-needle aspiration	0	0	118
DIA files	347	179	125
Risk stratification			
Low	98 (35.77%)	57 (34.34%)	62 (52.54%)
Intermediate	97 (35.40%)	68 (40.96%)	45 (38.14%)
High	79 (28.83%)	41 (24.70%)	11 (9.32%)
Sex			
Male	78	46	37
Female	196	120	81
Age (mean±SD)	45.55±13.70	45.68±13.68	46.06±13.26
Preoperative indices on ultrasonography			
Tumour diameter (mm)	14.21±8.49	12.17±8.47	11.41±8.96
Capsular invasion			
Yes	105	82	57
No	169	84	61
Extrathyroidal extension			
Yes	95	55	29
No	179	111	89
Multifocality			
Single focal	220	138	97
Multifocal	54	28	21
*BRAF*^V600E^ mutation			
Yes	161	52	39
No	34	5	24
Unknown	79	109	55

We collected clinical, immune, and genetic features and proteomic information. The preoperative clinical indicators included sex, age, tumour diameter (based on ultrasonography), Hashimoto’s thyroiditis (based on chemiluminescence detection and ultrasonography), multifocality (based on ultrasonography), capsular invasion (based on ultrasonography), and extrathyroidal extension (based on ultrasonography). Inflammatory conditions increase the risk of cancer, and systemic immune markers derived from the peripheral blood have been extensively investigated for diagnosis and prognosis in a variety of solid tumours, including thyroid carcinoma^24,25,33^. Here, we collected eight blood immunological indices before surgery: platelet counts, neutrophil counts, lymphocyte counts, monocyte counts, platelet-to-lymphocyte ratio, neutrophil-to-lymphocyte ratio, LMR, and SII. Since the *BRAF*^V600E^ mutation, which accounts for 60–70% of PTC mutations, is an independent predictor of the risk of recurrence^26–28^, we also detected the *BRAF*^V600E^ mutation using the amplification refractory mutation system (Supplementary Table S1, Supplemental Digital Content 1, http://links.lww.com/JS9/B246). For proteomics information collection, we applied PCT-DIA on a total of 558 specimens (FFPE slides or prospective FNA samples) from 558 PTC patients. To minimise the batch effects from the large sample sets, 274 PTC samples with replicates from the discovery set were randomly allocated into 22 batches and analysed by DIA-MS (Fig. 1B).

### Proteomic analysis

We analysed the DIA data using DIA-NN (v1.7.15) and our previously established thyroid library^30^. The library contains 157 548 peptide precursors, 121 960 peptides, 9941 protein groups, and 9826 proteins from proteotypic peptides. We identified and quantified 44 830 peptides and 5774 proteins (Supplementary Table S2 and Supplementary Table S3, Supplemental Digital Content 2, http://links.lww.com/JS9/B247, http://links.lww.com/JS9/B248). The CV of the pooled thyroid samples, correlation of the paired technical replicates, batch effect entity of the pooled samples, and correlation between biological duplicate samples are shown in Supplementary Figure S1 (Supplemental Digital Content 5, http://links.lww.com/JS9/B251). The results showed that the samples have good reproducibility.

**Figure 3:**
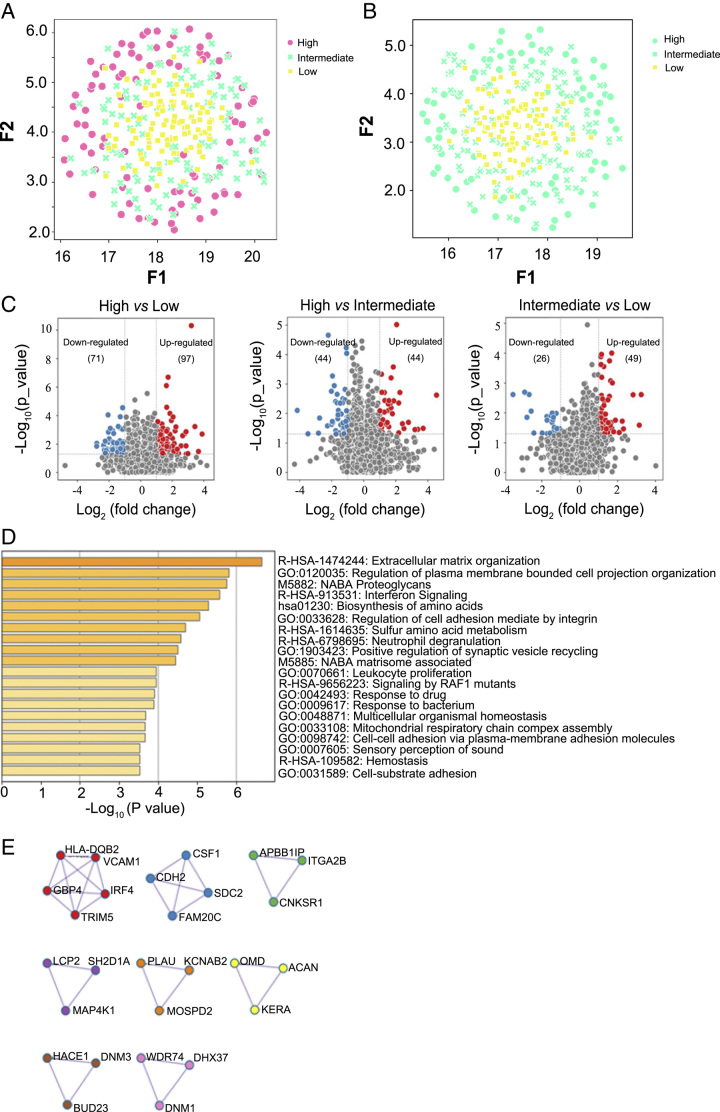
Proteomics analysis of the PTC samples. (A) UMAP plot showing the discrimination of the three groups of PTC samples under 5774 proteins. Each sample is colour-coded according to whether it belongs to a high-risk, intermediate-risk, or low-risk case (stratification performed postsurgically). (B) UMAP plot showing the discrimination of intermediate-/high-risk PTC samples and low-risk PTC samples. (C) Volcano plots showing the DEPs derived from the pairwise comparison of the high-risk, intermediate-risk, and low-risk groups, with the thresholds of |log_2_(fold change)| >1 and Welch’s *t* test *P*<0.05. (D) Biological function enrichment analysis of the DEPs derived from comparing the high-risk and low-risk groups through Metascape. (E) The MCODE algorithm was used to investigate the protein networks of the DEPs derived by comparing the high-risk and low-risk groups. Each MCODE network is assigned a unique colour.

We computed the average intensities of the 5774 proteins that were quantified with less than 90% missing values for each PTC. The UMAP plot exhibited the global view of the proteomic expression for the three groups. However, the intermediate-risk and high-risk samples could not be separated from each other, which implied similar biological characteristics between the two risk strata (Fig. 3A). We then grouped the intermediate-risk and high-risk samples, and the resulting cluster could be clearly separated from the low-risk samples in the UMAP analysis (Fig. 3B). This result is associated with the clinical treatment of PTC patients, in which low-risk PTC patients are usually recommended for active surveillance, while intermediate-risk and high-risk PTC patients are often treated with surgery and additional therapies. We then analysed the DEPs between the high-risk and low-risk groups. The abundances of 71 and 97 DEPs were decreased and increased, respectively, in the high-risk group relative to the low-risk group (unpaired two-sided Welch’s *t* test, *P*<0.05, |log_2_(fold change (FC)|>1) (Fig. 3C). We next performed pathway enrichment analysis based on the DEPs in the two groups. The results showed that signalling by *RAF1* mutants was involved in enrichment analysis and the Gene Ontology (GO) network, which suggested the critical role of the *BRAF*^V600E^ mutation in thyroid cancer from the proteomics perspective. In addition, signalling pathways related to tumour invasion, immune regulation, and amino acid biosynthesis or metabolism were found to be enriched in the high-risk group relative to the low-risk group (Fig. 3D, Supplementary Figure S2, Supplemental Digital Content 5, http://links.lww.com/JS9/B251). Using protein–protein interaction (PPI) analysis, we also identified eight protein clusters, including interferon signalling, post-translational protein phosphorylation, signalling by *RAF/BRAF* mutants, and TCR signalling, involved in the regulation of high-risk PTCs (Fig. 3E). These results showed that risk stratification of PTC can be carried out based on proteotypic maps, which provided an accurate depiction of PTC at different risk levels and further revealed crucial signalling pathways involved in PTC development.

### Feature selection and model development

**Figure 4:**
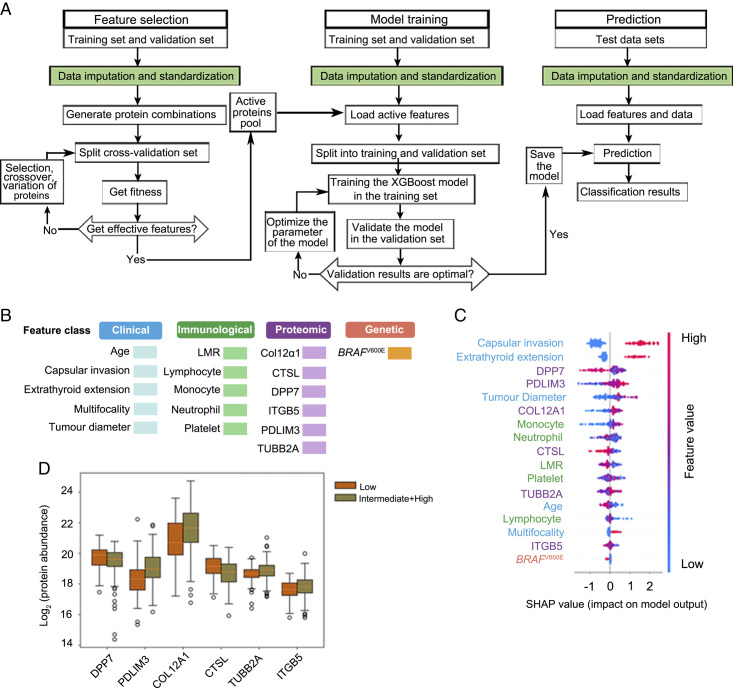
PRAC-PTC development and feature selection. (A) Workflow for feature selection, model training, prediction. (B) The 17 features were selected from four dimensions by XGBoost model. (C) Feature importance interpreted by SHapley Additive exPlanations (SHAP) algorithm. Red dots on the right indicate that the higher the expression of this feature, the more inclined it is to be predicted as intermediate-risk and high-risk. Red dots on the left indicate that the higher the expression of this feature, the more inclined it is to be predicted as low-risk. The higher the feature is, the higher the importance. (D) Protein expression plots for the six selected protein features using the discovery cohort in the intermediate-/high-risk cases *versus* the low-risk cases.

For subsequent analyses, the samples were divided into low-risk PTCs and intermediate-/high-risk PTCs. To derive a multidimension-based signature differentiating low-risk PTCs and intermediate-/high-risk PTCs, we developed a feature selection process combined with a machine learning model classifier based on the discovery set of 274 samples (Fig. 4A). We collected data on the clinical, immunological, genetic, and proteomic characteristics of PTC patients to build a model considering these four dimensions. We included 5790 variables from the four dimensions, and through different algorithms, we found that the model composed of 17 characteristic variables constructed with the XGBoost algorithm^34^ had the best predictive performance in separating low-risk and intermediate-/high-risk PTC and thus named it the Preoperative Risk Assessment Classifier for PTC (PRAC-PTC). The 17 variables included five clinical characteristics (age, capsular invasion, extrathyroid extension, multifocality, and tumour diameter), five blood immune indictors (LMR, lymphocytes, monocytes, neutrophils, and platelets), six proteins (collagen alpha-1[XII] chain [Col12A1], cathepsin L [CTSL], dipeptidyl peptidase 2 [DPP7], integrin subunit beta-5 [ITGB5], PDZ and LIM domain 3 [PDLIM3], and tubulin beta-2A class IIa [TUBB2A]) and one genetic feature (*BRAF*^V600E^ mutation) (Fig. 4B).

Furthermore, the importance values of the 17 variables used to construct PRAC-PTC were calculated by the SHapley Additive exPlanations (SHAP) algorithm^35^ (Fig. 4C). Out of the 17 characteristic variables, the SHAP algorithm identified two variables with the highest weights, both tumour invasion features: extrathyroidal extension and capsule invasion. Measurable with ultrasound, extrathyroidal extension, and capsule invasion are known to be high-risk characteristics of PTC and show predictive value in the recurrence of PTC^36,37^. We screened a total of six proteins out of 17 variables, of which three were associated with thyroid cancer and thyroid functions: PDLIM3^38^, CTSL^39^, and ITGB5^40^ (Table 2, Supplementary Table S4, Supplemental Digital Content 3, http://links.lww.com/JS9/B249). Another protein, Col12A1, has been reported to be only related to thyroid function. No previous association with thyroid disease has been reported for the remaining two proteins: DPP7 and TUBB2A. As depicted in Figure 4C, three proteins (including PDLIM3, Col12A1, and TUBB2A) tended to be highly expressed in intermediate-risk and high-risk PTC, and the three proteins (including DPP7, CTSL, and ITGB5) tended to be highly expressed in low-risk PTC. This result was further confirmed by the different patterns of the individual protein levels in the discovery set (Fig. 4D). These results revealed that these proteins are associated with the biological behaviour of PTC, indicating the importance of proteomics in PTC risk stratification. Among the five immune indices screened by PRAC-PTC, LMR was identified as an independent prognostic factor in high-risk PTC patients^41^. Neutrophil and platelet counts have also been reported to help discriminate PTC from benign thyroid disorders^42^. In addition, our model selected the *BRAF*^V600E^ mutation as another important feature, which corresponded to the invasive role of the *BRAF*^V600E^ mutation in PTC^43,44^.

**Table 2:** Six proteins selected by machine learning model and previously known associations with thyroid physiology or pathology.

Uniprot ID	Gene name	Protein name	Thyroid cancer related	Thyroid function related
Q99715	*COL12A1*	Collagen alpha-1(XII) chain	—	Yes
Q9UHL4	*DPP7*	Dipeptidyl peptidase 2	—	—
Q53GG5	*PDLIM3*	PDZ and LIM domain protein 3	Yes	Yes
P07711	*CTSL*	Cathepsin L	Yes	Yes
P18084	*ITGB5*	Integrin beta-5	Yes	Yes
Q13885	*TUBB2A*	Tubulin beta-2A chain	—	—

### Validation of PRAC-PTC

**Figure 5:**
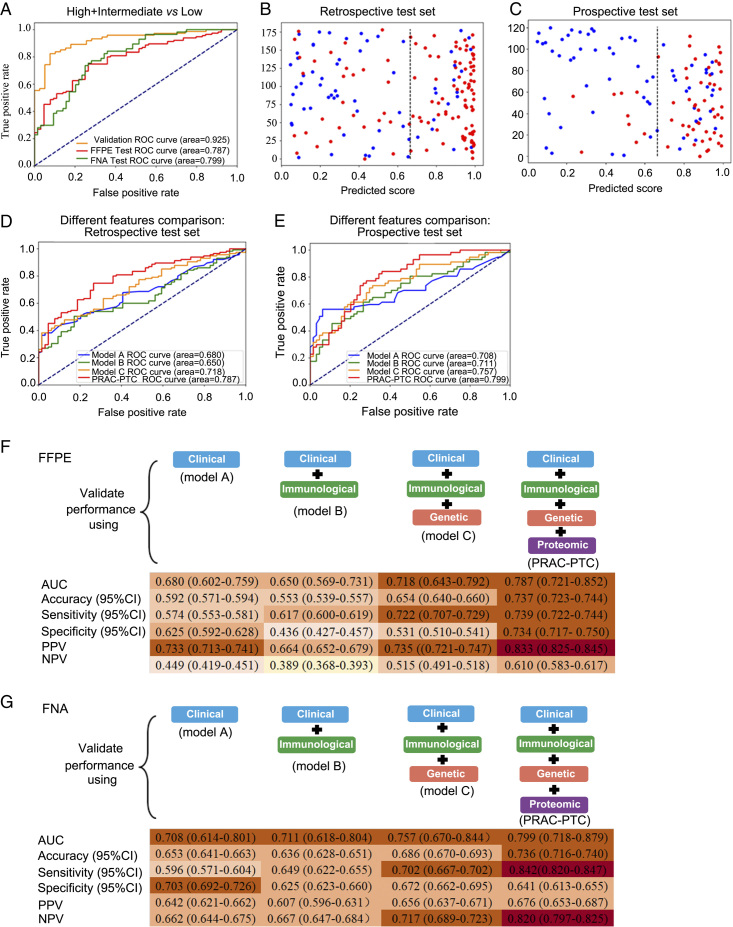
Classifier development, performance testing, and model validation using independent blinded datasets. (A) ROC plots of the model using the internal validation set, the FFPE test set, and the FNA test set. (B) Separation of the high-cases and intermediate-cases from the low-risk cases using PRAC-PTC and the retrospective test set. Blue plots represent low-risk PTC samples, and red plots represent intermediate-risk and high-risk PTC samples. (C) Separation of the high-cases and intermediate-cases from the low-risk cases using PRAC-PTC and the prospective test set. Blue dots represent low-risk PTC samples, and red dots represent intermediate-risk and high-risk PTC samples. (D) ROC plots showing the performance of XGBoost models with different dimensional features. The performance was tested using the prospective test set. (E) ROC plots showing the performance of XGBoost models with different dimensional features. The performance was tested using the retrospective test set. (F) The diagnostic performance of XGBoost models with different dimensional features, assessed on the FFPE test set. (G) The diagnostic performance of XGBoost models with different dimensional features, assessed on the FNA test set.

We next validated PRAC-PTC in risk stratifying PTC patients. Using PRAC-PTC, each specimen among the 191 randomly selected samples from the discovery set (70%) used for model training was reclassified into low-risk or intermediate-/high-risk. We validated this model on the remaining 83 samples (30% of the discovery set) from the same cohort. The XGBoost algorithm achieved area under the curve (AUC) values of 0.925 for the internal validation set (*n*=274) (Fig. 5A). To validate PRAC-PTC in independent cohorts, we first analysed 166 pathologist-reviewed FFPE PTCs. Receiver operating characteristic curve (ROC) analysis in this retrospective independent test set using the PRAC-PTC showed an AUC of 0.787 (95% CI: 0.721–0.852) (Fig. 5A). We further extended the validation to an independent prospective test set composed of the preoperative FNA biopsy samples to demonstrate its clinical applicability value. The FNA test set comprised 118 FNA samples from 118 patients, all of whom underwent thyroidectomy after preoperative FNA. Using initial postoperative histopathological diagnoses of thyroid tumour tissues as the benchmark, our model achieved an AUC value of 0.799 (95% CI: 0.718–0.879) (Fig. 5A). Scatter plots show distinct separation between low-risk and intermediate-/high-risk PTCs in the FFPE and FNA test sets (Fig. 5B–C).

Furthermore, we utilised the clinical, immune, and genetic information that was conveniently obtained from preoperative examinations for modelling to evaluate their predictive performance. First, we built three models (model A, model B, and model C) based on an increasing number of feature dimensions to predict the risk degree of PTC in the retrospective FFPE test set. Model A was built based on clinical information alone, model B was built based on both clinical and immune information, and model C was built based on clinical, immune, and genetic information (Fig. 5D, F). The AUC values were 0.680 (95% CI: 0.602–0.759) for model A and 0.650 (95% CI: 0.569–0.731) for model B. When the *BRAF*^V600E^ mutation was included as the third feature dimension, the AUC value increased to 0.718 (95% CI: 0.643–0.792) (model C). Compared to models A, B, and C, the AUC values of PRAC-PTC were greater by 15.7, 21.1, and 9.6%, respectively (*P*-value <0.05 for all). The performance was also tested in the prospective FNA test set, and the AUC values of PRAC-PTC were 12.9, 12.4, and 5.5% greater (*P*-value <0.05 for the above three), than those of models A, B, and C, respectively (Fig. 5E, G). These results highlight the indispensable role of proteomics in risk stratification, as its features provided the most accurate predictions when combined with clinical, immune, and gene features.

### Evaluation of the effectiveness of PRAC-PTC

**Figure 6:**
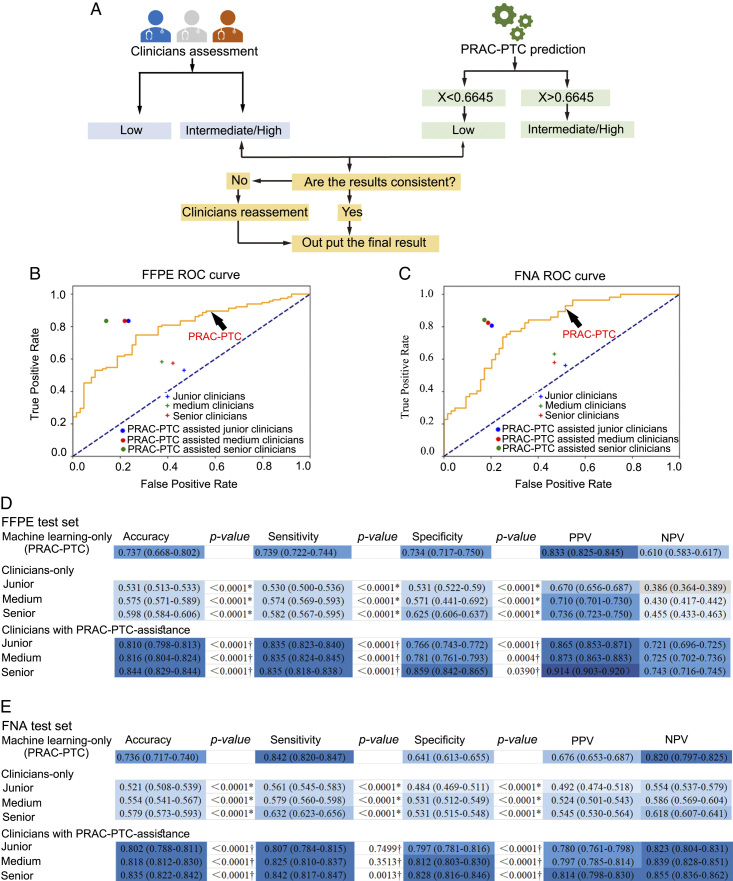
Evaluation performance of the PRAC-PTC, clinicians and clinicians assisted with the PRAC-PTC for the discrimination of low-risk PTC from intermediate-/high-risk PTC. (A) Evaluation performance of the PRAC-PTC, clinicians only and clinicians assisted with the PRAC-PTC in the FFPE test sets. The ROC plot shows the performance of the machine learning model with four-dimensional features. Dots indicate the sensitivities and specificities of clinicians with different seniority levels. Cruciate pixels indicate the sensitivities and specificities of clinicians assisted with the machine learning model. (B) Evaluation performance of the PRAC-PTC, clinicians only and clinicians assisted with the PRAC-PTC in the FNA test set. The ROC plot shows the performance of the machine learning model with four-dimensional features. Dots indicate the sensitivities and specificities of clinicians with different seniority levels. Cruciate pixels indicate the sensitivities and specificities of clinicians assisted with the machine learning model. (C) The diagnostic performance of PRAC-PTC, clinicians alone, and PRAC-PTC-assisted clinicians in retrospective test set. (D) The diagnostic performance of PRAC-PTC, clinicians alone, and PRAC-PTC-assisted clinicians in prospective test set. F1, a weighted average of the PPV and sensitivity; NPV, negative predictive value; PPV, positive predictive value; *Performance of PRAC-PTC with Clinicians only; †Performance of machine learning-only with Clinicians assisted with PRAC-PTC.

Furthermore, we investigated the application of PRAC-PTC in clinical practice. First, we observed the accuracy of the nine clinicians with different seniority levels, including three junior clinicians (1–5 years of experience), three medium-level clinicians (5–10 years of experience), and three senior clinicians (>10 years of experience), in preoperatively predicting the risk of PTC based on ultrasound and CT (Supplementary Figure S3, Supplemental Digital Content 5, http://links.lww.com/JS9/B251). Detailed information on the clinicians’ preoperative risk stratification process is shown in the Methods section and Figure 6A. Second, the PRAC-PTC outputs a score for each patient; if the score is less than 0.6645, the PRAC-PTC considers the case to be low-risk PTC; if the score is more than 0.6645, the PRAC-PTC considers the case to be intermediate-/high-risk PTC. Third, initial independent assessments were made by the nine clinicians alone and then with assistance from PRAC-PTC for the final assessments. If the two results did not match, the clinicians could then choose either their own result or PRAC-PTC’s result as the final result (Supplementary Table S5, Supplemental Digital Content 4, http://links.lww.com/JS9/B250). We compared the effectiveness of PRAC-PTC and the clinicians with different seniority levels (Fig. 6B–C). The predictive performance (accuracy, sensitivity, specificity, NPV, and PPV) gradually improved with increasing clinician seniority (junior-medium-senior) in the retrospective and prospective test sets (Fig. 6D–E). In the retrospective test set, the predictive performance of PRAC-PTC [73.7% (95% CI: 66.8–80.2) for accuracy, 73.9% (95% CI: 72.2–74.4) for sensitivity, 73.4% (95% CI: 71.7–75.0) for specificity] was higher than that of senior clinicians only [59.8% (95% CI: 58.4–60.6) for accuracy, 58.2% (95% CI: 56.7–59.5) for sensitivity, 62.5% (95% CI: 60.6–63.7) for specificity, *P*<0.0001] (Fig. 6D). In the prospective test set, the performance of PRAC-PTC [73.6% (95% CI: 71.7–74.0) for accuracy, 84.2% (95% CI: 82.0–84.7) for sensitivity, 64.1% (95% CI: 61.3–65.5) for specificity] was also significantly higher than that of senior clinicians only [57.9% (95% CI: 57.3–59.3) for accuracy, 63.2% (95% CI: 62.3–65.6) for sensitivity, 53.1% (95% CI: 51.5–54.8) for specificity, *P*<0.0001] (Fig. 6E). These results showed the predictive superiority of PRAC-PTC over the clinicians.

We further explored the ability of the clinicians to evaluate cases with assistance from PRAC-PTC. PRAC-PTC-assisted senior clinicians achieved better predictive performance than PRAC-PTC alone. In the retrospective test set, the accuracy of PRAC-PTC was 73.7% (95% CI: 66.8–80.2), which improved to 84.4% (95% CI: 82.9–84.4) when senior clinicians used its results (*P*<0.0001; Fig. 6B, D). In the prospective test set, the accuracy of the PRAC-PTC was 73.6% (95% CI: 71.7–74.0), which improved to 83.5% (95% CI: 82.2–84.2) for senior clinicians assisted with PRAC-PTC (*P*<0.0001; Fig. 6C, E). We also found that the performance of PRAC-PTC-assisted junior clinicians was greatly improved. For the junior clinicians, PRAC-PTC assistance increased the sensitivity and specificity by 57.5 and 44.3% in the retrospective test set and 43.9 and 64.7% in the prospective test set, respectively (Fig. 6D–E). These results showed that the preoperative predictive risk effectiveness of clinicians was improved with the assistance of the new classifier.

## Discussion

Recent advancements have emerged in the diagnosis of papillary thyroid carcinoma. One such example is the combination of FNA and gene detection guided by ultrasonography, which has the potential to enhance the diagnostic efficacy for PTC. However, there remains a dearth of effective preoperative risk assessment methods for PTC patients. In this study, we constructed a machine learning model utilising multidimensional features to predict the risk level of PTC patients. We generated a model named PRAC-PTC and independently validated it in internal, external retrospective, and external prospective cohorts. Furthermore, we found that clinicians significantly improved their ability to assess the risk degree of preoperative PTC with the assistance of PRAC-PTC.

Some clinical features of PTC are commonly used by clinicians to predict the risk degree. However, the accuracy of such evaluations is influenced by various objective factors, including the interference caused by the tracheoesophageal groove on ultrasound images, as well as subjective factors such as the expertise of the clinicians involved. For instance, the evaluation of lymph node metastasis as an indicator of PTC risk is challenging due to difficulties in accurately determining the size and number of metastatic lymph nodes. Thus, relying solely on clinical characteristics may make assessing the degree of risk in PTC challenging. In this study, the AUC of the model built from clinical features alone was only 0.680 (95% CI: 0.602–0.759). Given the close relationship between tumour development and the immune status of individuals^45^, previous studies attempted to predict the biological behaviour of thyroid tumours by examining immune indicators in the blood^46–48^. In our recent study, we identified tumour diameter and SII as independent risk factors for lateral lymph node metastasis in PTC^49^. Consequently, this study incorporates commonly used blood immune indicators as a crucial predictive dimension. We found that five out of eight blood immune indictors were associated with PTC risk. Similarly, the identification of appropriate genetic markers to help stratify PTC risk is also a great challenge in this field. Although the *BRAF*^V600E^ mutation is the main genetic driver for the occurrence and development of PTC, its correlation with the biological characteristics of PTC is controversial^50^. This study showed a correlation between the *BRAF*^V600E^ mutation and PTC risk, albeit with a relatively small weight, as the mutation was ranked last among the 17 variables considered.

Proteomics has been widely applied to clinical outcome prediction as a highly refined molecular prediction biomarker^51–53^. In some studies, proteomic analysis has demonstrated superior predictive performance compared to clinical models constructed solely based on clinical information. In our study, we employed a data-independent acquisition (DIA) mode with mass spectrometry to conduct proteomic profiling and successfully identified and quantified 5774 proteins associated with PTC. The protein profile provided an accurate description of PTC at different risk levels. By employing the XGBoost algorithm, we selected six proteins to distinguish low-risk PTCs from intermediate-/high-risk PTCs. Among these proteins, three exhibited significant weights in the model and emerged as key quantitative indicators for assessing the degree of PTC risk. Furthermore, we found that the combination of four-dimensional features yielded the optimal predictive performance. This finding suggests that each evaluation method contributes uniquely, and when single-dimension predictions fail to achieve satisfactory results, expanding the dimensions of analysis may present a more effective solution.

The management plan for PTC is determined by clinicians, and to aid in this process, we developed the PRAC-PTC tool. In clinical practice, PRAC-PTC outputs a score for each patient; if the score is less than 0.6645, PRAC-PTC considers the case to be low-risk PTC, and the patient can temporarily avoid surgery and undergo active surveillance. If the score is more than 0.6645, PRAC-PTC considers the case to be intermediate-/high-risk PTC, indicating that the patient needs more active treatment, such as surgery or surgery and I^131^ treatment. We observed significant differences in the accuracy of assessment of PTC risk by clinicians with different seniority levels due to their subjective experience. PRAC-PTC, demonstrating the ability to objectively quantify the risk of PTC patients, exhibited greater predictive potential than the clinicians. When utilised in an assistive capacity, PRAC-PTC significantly enhanced the predictive capabilities of clinicians across all experience levels, with the most substantial improvement observed among junior clinicians. The PRAC-PTC-assisted senior clinicians showed the best evaluation performance, with 84.4 and 83.5% predictive accuracy in the retrospective and prospective test sets, respectively. In view of the continuous growth of PTC incidence, the application of PRAC-PTC not only guides accurate management but also facilitates the reasonable allocation of medical resources.

Notably, there are still some inherent biases in the application of PRAC-PTC. One of the current limitations is that the model is currently only applicable to Asians. The sample size needs to be expanded, and the number of centres needs to be increased. Validation with independent centre data from more countries would contribute to testing the model’s generalisation ability. In addition, we used nontargeted proteomics testing to detect potential protein biomarkers. In subsequent validation processes, we will further detect these proteins through faster, less expensive, and more stable targeted proteomics or antibody methods. Moreover, the acquisition of thyroid proteomics relies on FNA, which is an invasive procedure that limits its widespread application to some extent. Therefore, we will consider applying body fluid samples in further research.

We consider there to be several potential research directions in this field. First, the predictive performance of PRAC-PTC is not ideal. In future research, we will use multiomics sequencing and multimodal data integration to stratify PTC risk to achieve better predictive performance. Second, given the differences in surgical approaches and postoperative management between intermediate-risk and high-risk PTC cases, further subdivision of these risk categories is warranted in future studies. Third, we focused on C-PTC in this study, and the risk stratification of other types of thyroid cancer should also be explored in future studies.

In conclusion, this study represents the first demonstration of the effective utility of PRAC-PTC, which employs multidimensional features for assessing the risk of PTC. PRAC-PTC not only provides valuable guidance for clinical decision-making but also contributes to a reduction in unnecessary surgical interventions. The convenience of obtaining and applying key preoperative information from four dimensions renders PRAC-PTC highly applicable in clinical practice, suggesting its potential for delivering a significant clinical impact.

## Ethical approval

The Helsinki Declaration and any updates or similar ethical standards, as well as institutional and/or national research committee ethical standards, were followed in all procedures employed in the research involving human beings. Ethical approval for this study (2019-039-01) was provided by the Ethical Committee of Hangzhou First People’s Hospital.

## Consent

All patients agreed to participate in this study and provided informed consent. Written informed consent was obtained from the patient for publication of this research and any accompanying images. A copy of the written consent is available for review by the Editor-in-Chief of this journal on request.

## Sources of funding

This work was supported by grants from the National Key R&D Programme of China (2022YFF0608403) to Yi Zhu, National Key R&D Programme of China (Nos. 2021YFA1301600, 2020YFE0202200) to Tiannan Guo, Zhejiang Basic Public Welfare Research Plan Project (GF22H165705), Zhejiang Provincial Basic Public Welfare Research Project (LGF22H160082), Zhejiang Medical and Health Science and Technology Plan Project (2022KY939) and Hangzhou Medical and Health Science and Technology Major Project (Z20210025) to Dingcun Luo, China Postdoctoral Science Foundation (2022M722841) to Yaoting Sun, and Zhejiang Medical and Health Science and Technology Plan Project (2023RC218) to Yuanhui Li.

## Author contribution

Y.L.: writing – original draft, visualisation, writing – review and editing, formal analysis; F.W.: investigation, data curation, and validation; W.G.: data curation, methodology, software, and validation; Y.Z.: conceptualization, investigation, and resources; Y.H.: data curation, software, and validation; L.Z.: investigation and visualisation; W.G.: software and validation; J.S.: investigation and resources; Y.N.: investigation, resources, and formal analysis; L.L.: resources and software; W.F.: data curation and formal analysis; X.L.: investigation and data curation; Y.Y.: formal analysis; Z.H.: investigation and methodology; C.C.: investigation and project administration; R.X.: investigation; S.Z.: resources; L.Z.: investigation; G.P.: resources; Y.P.: methodology; L.M.: validation; T.Z.: investigation; J.Z.: formal analysis; H.Z.: resources and supervision; Y.S.: data curation, formal analysis, methodology, and supervision; T.G.: conceptualization, data curation, funding acquisition, supervision, and project administration; D.L.: conceptualization, data curation, funding acquisition, supervision, project administration, and writing – review and editing.

## Conflicts of interest disclosure

T.G. is a shareholder of Westlake Omics Inc. W.G., W.F. and Y.H. are employees of Westlake Omics Inc. The other authors declare no competing interests in this paper. D.L, Y.L, F.W, Y.Z, J.S, Y.S. and T.G. have applied for two patents on this project.

## Research registration unique identifying number (UIN)

1. Name of the registry: Research Registry.
2. Unique identifying number or registration ID: ChiCTR2300069839.
3. Hyperlink to your specific registration: https://www.chictr.org.cn/bin/project/edit?pid=56056.

## Guarantor

Prof. D.L. accept full responsibility for the work.

## Data availability statement

The datasets used and analysed during the current study are available from the corresponding author on reasonable request.

## Provenance and peer review

Not commissioned, externally peer-reviewed.
